# Using Structural Equation Modeling to Understand Interactions Between Bacterial and Archaeal Populations and Volatile Fatty Acid Proportions in the Rumen

**DOI:** 10.3389/fmicb.2021.611951

**Published:** 2021-06-09

**Authors:** Veronica Kaplan-Shabtai, Nagaraju Indugu, Meagan Leslie Hennessy, Bonnie Vecchiarelli, Joseph Samuel Bender, Darko Stefanovski, Camila Flavia De Assis Lage, Susanna Elisabeth Räisänen, Audino Melgar, Krum Nedelkov, Molly Elizabeth Fetter, Andrea Fernandez, Addison Spitzer, Alexander Nikolov Hristov, Dipti Wilhelmina Pitta

**Affiliations:** ^1^Department of Clinical Studies, School of Veterinary Medicine, University of Pennsylvania, Kennett Square, PA, United States; ^2^Department of Animal Science, The Pennsylvania State University, University Park, PA, United States

**Keywords:** dairy cows, host-microbe interactions, inter-species hydrogen transfer, metabolically- active microbes, microbial syntrophy, rumen microbiota

## Abstract

Microbial syntrophy (obligate metabolic mutualism) is the hallmark of energy-constrained anaerobic microbial ecosystems. For example, methanogenic archaea and fermenting bacteria coexist by interspecies hydrogen transfer in the complex microbial ecosystem in the foregut of ruminants; however, these synergistic interactions between different microbes in the rumen are seldom investigated. We hypothesized that certain bacteria and archaea interact and form specific microbial cohorts in the rumen. To this end, we examined the total (DNA-based) and potentially metabolically active (cDNA-based) bacterial and archaeal communities in rumen samples of dairy cows collected at different times in a 24 h period. Notably, we found the presence of distinct bacterial and archaeal networks showing potential metabolic interactions that were correlated with molar proportions of specific volatile fatty acids (VFAs). We employed hypothesis-driven structural equation modeling to test the significance of and to quantify the extent of these relationships between bacteria-archaea-VFAs in the rumen. Furthermore, we demonstrated that these distinct microbial networks were host-specific and differed between cows indicating a natural variation in specific microbial networks in the rumen of dairy cows. This study provides new insights on potential microbial metabolic interactions in anoxic environments that have broader applications in methane mitigation, energy conservation, and agricultural production.

## Introduction

Anaerobic microbial ecosystems are ubiquitous on the planet, contributing significantly to the recycling of nutrients such as carbon, nitrogen, and sulfur (Whitman et al., 1998; Mori and Kamagata, 2014), and play important roles in deconstruction of organic biomass, the global carbon cycle, agricultural production, and the health of animals and humans. Under energy-constrained conditions, anaerobic microorganisms exchange metabolic products for mutual benefit to survive and maintain a stable microbial community structure (Lovley, 2017). Cooperation among microbes for mutual benefit, often referred to as obligatory metabolic mutualism or microbial syntrophy, is critical for the functionality of anaerobic microbial ecosystems and therefore has ecological significance (Morris et al., 2013). A known concept of microbial syntrophy, established in pure and mixed cultures, is the obligatory mutualism reported between bacteria and archaea for metabolic hydrogen (H_2_). This syntrophy, in which carbon is ultimately reduced to methane (CH_4_), is the hallmark of most anaerobic environments such as those found in freshwater sediments, swamps, paddy fields, landfills, and the intestinal tracts of ruminants and termites (Morris et al., 2013; Pitta et al., 2018). As much as a gigaton of CH_4_ is released from the turnover of 2% of atmospheric carbon dioxide (CO_2_) via biomass degradation through these anaerobic environments (Thauer et al., 2008). Therefore, a better understanding of metabolic interactions in microbial communities may give insights into complex processes at the host or habitat level. While there have been several theoretical and empirical reports on microbial syntrophy, information on the presence of microbial metabolic interactions in anaerobic microbial ecosystems, their functional relevance, how they change in temporal scales, and whether there is a natural selection for specific microbial metabolic interactions in anoxic environments is not completely understood.

Ruminants possess a complex rumen microbial ecosystem (RME), comprised of bacteria, protozoa, fungi, and archaea in the reticulorumen that work synergistically to ferment feed (Pitta et al., 2018). Bacteria, protozoa, and fungi release H_2_ during fermentation of feed, which must be transferred to different acceptors to allow re-oxidization of reduced cofactors (Ungerfeld, 2015). Methanogenic archaea, owing to their low thresholds for H_2_, capture most of this H_2_ and allow for homeostasis and continued feed fermentation in the rumen. Methane formation via methanogenesis in methanogenic archaea represents the final step of organic matter fermentation in anaerobic environments (Evans et al., 2019). Different methanogens such as *Methanobrevibacter*, *Methanosphaera*, and *Methanomassiliicoccaceae*, capable of reducing CO_2_, methanol, and methylamines, respectively (Boone et al., 1993), are known to exist in the rumen. Although species of *Methanobrevibacter* are predominant in the rumen, it has been demonstrated that *Methanosphaera* and *Methanomassiliicoccales* representatives may be more metabolically active than *Methanobrevibacter* species and may contribute significantly more to total methanogenesis than previously thought (Söllinger et al., 2018; Söllinger and Urich, 2019). However, the distribution of methanogens, their contribution to methanogenesis, and how they interact with H_2_-producing bacteria is largely unknown. As it has been established that the requirements and thresholds for H_2_ are much lower for methanol- and methylamine-utilizing methanogens such as *Methanosphaera* and *Methanomassiliicoccales* compared to CO_2_-reducing *Methanobrevibacter* species (Thauer et al., 2008), we hypothesized that different methanogens form interactions with specific bacteria resulting in different potential microbial metabolic networks in the rumen. Because microbes constantly interact among themselves and also interact with both the host and environmental factors, the rumen can serve as an excellent model for unraveling specific microbial metabolic associations and also for the development of prediction models on microbial syntrophic networks.

To identify specific bacteria-archaea networks, we sought to explore potential metabolic bacteria-archaea interactions within the RME of dairy cows. Because sampling time, sampling methods, and nucleic acid extraction (DNA-based vs. RNA-based analysis) can influence microbial community composition, we considered the possible factors from sample collection, processing, and analysis to reduce the bias introduced by these factors and to enable us to identify the significant potential microbial metabolic networks in the rumen from a cohort of dairy cows that were adapted to a standard diet. Further, our intent was not only to identify specific potential microbial metabolic associations but also to determine the causalities of these networks by fitting the identified causalities into a validated hypothesis-driven prediction model (Rabe-Hesketh et al., 2004; Mamet et al., 2019).

This study demonstrates the presence of possible metabolic associations of bacteria-archaea cohorts in the rumen that are cohort-specific but different between cohorts of dairy cows. Our data provide detailed and novel insights on potential microbial metabolic networks linked to metabolic end products. We also employed a multivariate statistical analysis technique to assess and quantify the interactions. Findings from this project are broadly applicable to understanding ecological microbial metabolic interactions underlying community composition and the mechanistic basis of anerobic microbial ecosystems.

## Materials and Methods

### Animal Handling and Rumen Sampling

The Pennsylvania State University Animal Care and Use Committee approved all animal-related procedures used in this experiment. Six ruminally cannulated lactating Holstein cows adapted to a standard silage-based diet were enrolled in the experiment, which consisted of 17 days of adaptation followed by 2 days of sampling.

### Milk Yield Measurements, Milking and Feeding Information

Milk yield was recorded daily throughout the experiment and averaged (±SD) 49.8 ± 9.68 kg/day. The cows averaged 2.20 ± 0.37 lactations, 565 ± 40.9 kg BW, and 45.9 ± 11.7 DIM at the beginning of the experiment. All animals were milked twice daily at 0600 and 1800 h and had free access to drinking water. Before morning milking the cows were kept in an exercise area for 1 h. They were fed once daily (at 0800 h) a standard diet containing (DM basis): 36.8% corn silage, 15.2% alfalfa haylage, 13.6% ground corn grain, 8.8% canola meal, 8.0% cookie meal, 5.6% roasted soybean seeds, 4.8% molasses, 3.2% whole cottonseed, 2.0% grass hay, 1.8% vitamin and mineral premix, and 0.2% slow-release urea (Optigen^®^, Alltech Inc., Nicholasville, KY, United States).

### Sampling of Rumen Contents

Rumen samples were collected via both stomach tube and rumen cannula to assess whether potential microbial metabolic associations could be detected in both ruminal sample types. Sampling was completed at 0, 2, 4, 6, 8, and 12 h after the morning feeding divided across 2 consecutive days. A total of 216 samples, inclusive of all sampling types, were collected for DNA and RNA extraction. For sampling of rumen contents via cannula (hereafter referred to as CS samples), the cannula lid was removed and whole rumen content samples were collected from 4 locations in the rumen. The sample collection sites were the ventral sac, the atrium or reticulum, and two samples from the feed mat, and following collection, these were combined into one CS sample. Approximately 500 g of ruminal contents were removed during each sampling event. For sampling of rumen contents via stomach tubing (hereafter referred to as TS samples), the head of the animal was restrained, and rumen fluid was collected by passing an orogastric tube (244 cm long) using an oral speculum as described in Pitta et al. (2014). Approximately 100–200 ml of initially sampled rumen digesta was discarded due to possible salivary contamination. The subsequent rumen samples retrieved by stomach tube (200–250 ml) or cannula were separated into liquid and solid fractions by filtering through four layers of cheesecloth, to obtain at least 25 g of ruminal solids. About 25 g of the sample was frozen and archived for DNA analysis. About 2 g of sample was transferred to tubes containing 5 ml of TRIzol, frozen and archived for RNA analysis. Subsamples of the cheesecloth filtrates were processed for analysis of volatile fatty acids (VFAs) as described in Hristov et al. (2011). Briefly, subsamples of 45-mL of the cheesecloth filtrate were centrifuged at low speed (500 × *g*, or 1,700 × rpm for 5 min at 4°C) to remove protozoa and feed particles. A 5-mL aliquot of the low-speed supernatant was combined with 1 mL of 25% metaphosphoric acid and 1 mL of 0.6% 2-ethyl-butyric acid, as internal standard solution, centrifuged at 20,000 × *g* for 15 min at 4°C, and then kept frozen (−20°C) until used for VFA analysis. VFAs were analyzed using gas-liquid chromatography with a column 2 m long × 2 mm in diameter packed with Carbowax 20 M on 80/100 Carbopack BPA (Supelco, Inc., Bellefonte, PA, United States).

### Genomic DNA Extraction, RNA Extraction, and PCR Amplification

The genomic DNA from all samples was extracted using the repeated bead-beating and column (RBB + C) method of DNA extraction (Yu and Morrison, 2004) followed by extraction with a commercial kit (Qiagen QIAamp Fast DNA Stool Mini Kit; Qiagen Sciences, Germantown, MD, United States). RNA extraction from all samples was performed using a modified version of the acid guanidinium thiocyanate-phenol-chloroform (TRIzol) method (Chomczynski and Sacchi, 1987; Li et al., 2016) as per the method described in Li et al. (2016). Briefly, ∼250 mg of TRIzol-preserved sample was bead-beaten in 1 mL of fresh TRIzol, then precipitated using 0.2 mL chloroform and 0.5 mL isopropanol, washed in 1 mL 80% ethanol, and eluted in molecular-biology grade water. A NanoDrop 2000 (Thermo Fisher Scientific, Waltham, MA, United States) was used to assess RNA quality and quantity. In addition, a Bioanalyzer (Agilent Technologies, Santa Clara, CA, United States) was used to determine the RNA integrity number (RIN) of a selected set of samples, with a high RIN number considered to be a marker of high-quality RNA. Following extraction for RNA, samples were converted to cDNA using the SuperScript VILO cDNA Synthesis Kit (Invitrogen, Carlsbad, CA, United States) according to the manufacturer’s instructions.

For each extracted genomic DNA and cDNA sample, the V1–V2 region of the bacterial 16S rRNA gene and the V6–V8 region of the archaeal 16S rRNA gene were PCR-amplified in triplicate using bacterial- and archaeal-specific primers barcoded with a unique 12-base error-correcting Golay code for multiplexing (Song et al., 2013). Polymerase chain reaction was performed using the Accuprime Taq DNA Polymerase System (Invitrogen, Carlsbad, CA, United States).

The bacterial-specific primers used were F27 (5′-AGAGTTTGATCCTGGCTCAG-3′) and R338 (5′-TGCTGCCTCCCGTAGGAG T-3′), and the archaeal-specific primers used were i958aF (5′-AATTGGAKTCAACGCCKGR-3′) and i378aR (5′-TGTGTGCAAGGAGCAGGGAC-3′). The thermal cycling conditions for PCR amplification of the bacterial 16S rRNA gene involved an initial denaturing step at 95°C for 5 min followed by 20 cycles (denaturing at 95°C for 30 s, annealing at 56°C for 30 s, extension at 72°C for 90 s) and a final extension step at 72°C for 8 min. The thermal cycling conditions for PCR amplification of the archaeal 16S rRNA gene involved an initial denaturing step at 94°C followed by 30 cycles (denaturing at 94°C for 30 s, annealing at 56 for 1 min 30 s, extension at 72°C for 30 s) and a final extension step at 72°C for 8 min. The triplicate amplicon products from each sample were pooled and then quantified using a Spectramax M2^*e*^ microplate reader (Molecular Devices, San Jose, CA, United States). The quantified amplicons were combined by adding each sample to bacterial and archaeal pools in equimolar concentration. The final pool was bead purified using Beckman Coulter Agencourt AMPure XP Beads (Beckman Coulter, Brea, CA, United States).

### Sequencing, Data Analysis, and Statistical Analysis

Sequencing was performed at the PennCHOP Microbiome Core, University of Pennsylvania, using the Illumina MiSeq platform. The bacterial sequences were processed through the QIIME2 (2018.4) pipeline (Bolyen et al., 2019). The sequence reads were de-multiplexed and assigned to amplicon sequence variants (ASV) using the DADA2 plugin (Callahan et al., 2016). Multiple sequence alignment was carried out with MAFFT (version 7; Katoh, 2002) and sequences were filtered to remove highly variable positions. FastTree 2 (version 2; Price et al., 2010) was used to construct and root a phylogenetic tree. Taxonomic classification was conducted using a pre-trained Naive Bayes classifier trained on the Greengenes database (12/10 release; McDonald et al., 2012) for the V1–V2 region of the 16S rRNA gene (DeSantis et al., 2006). The archaeal reads were analyzed using the QIIME 1.8.0 pipeline (Caporaso et al., 2010). The paired end sequences were merged, de-multiplexed, and quality filtered. The operational taxonomic units (OTU) were formed by clustering sequences based on a 97% similarity threshold using the UCLUST algorithm (version 1.2.22; Edgar, 2010). Representative sequences for each OTU were aligned with PyNAST (version 1.2.2; Caporaso et al., 2010). The resultant multiple sequence alignment was used to infer a phylogenetic tree with FastTree 2 (version 2; Price et al., 2010). The taxonomy of each archaeal sequence was identified using the UCLUST consensus taxonomy assigner by performing a search against GreenGenes taxonomy (12/10 release; McDonald et al., 2012). Alpha diversity was assessed via observed species and Shannon diversity and beta diversity was measured using weighted and unweighted UniFrac distances. The measured alpha diversity matrices were compared between the treatment groups using the Wilcoxon/Kruskal–Wallis Rank Sum Test. For beta diversity matrices, a non-parametric permutational multivariate ANOVA test (Anderson, 2001), implemented in the vegan package for R, was used to test the interactions and main effects. We calculated Bray Curtis dissimilarity indices for VFA analysis using the “vegdist” function available in the R vegan package.

The raw read counts from the 16S rRNA ASV abundance table were collapsed at taxonomic rank and compositionally normalized (relative abundance) such that each sample summed to 1. To test the differences in bacterial/archaeal taxa between treatment groups, the Analysis of Composition of Microbiomes (ANCOM) (Mandal et al., 2015) procedure available in R was used. The significance of test was determined using the Benjamini-Hochberg procedure that controls for false discovery rate. A *P* value of <0.05 was used to define significance. Spearman correlation was used to correlate VFA parameters with OTU of the abundant genera using R. Genera were considered present in a sample if their sequence proportion was at least 0.01% of relative abundance.

### Structural Equation Modeling

Inference statistical analysis was performed using Structural Equation Modeling (SEM) implementation in STATA 16MP (College Station, TX, United States). In contrast to hypothesis-free genome wide scans, SEM is a hypothesis-driven approach (Rabe-Hesketh et al., 2004). Here, we assumed that the effect of methanogenic archaea modulates the endogenous production of VFA via “mediation” variables, which in our case were the H_2_-producing bacteria that have previously been associated with specific archaea and VFA. Since the SEM methodology requires a large number of samples, we maintained the simplest models possible. *Post hoc* analysis was used to estimate the goodness-of-fit of the model and also to estimate the indirect and total effects. We used two-sided tests of hypotheses and a *P* value < 0.05 as the criterion for statistical significance.

## Results

### Sequencing Details

For bacterial communities, approximately 12 million raw partial 16S rRNA sequences were obtained from 142 samples (DNA: 71 and cDNA: 71), with an average of 90,707 reads per sample and a range of between 15,444 (min) and 132,716 (max) reads. A total of 28,873 ASV (Supplementary Table 1A) were produced. For archaeal communities, approximately 3 million raw reads were obtained from 94 samples (DNA: 47 and cDNA: 47), with an average of 32,794 reads per samples and a range of between 51,200 and 19,583 reads. A total of 5,764 OTU (Supplementary Table 1B) were produced. Less than 200 reads per sample were observed in the PCR blank samples of bacterial and archaea.

### Bacterial and Archaeal Microbial Communities in the Rumen of Dairy Cows

**FIGURE 1:**
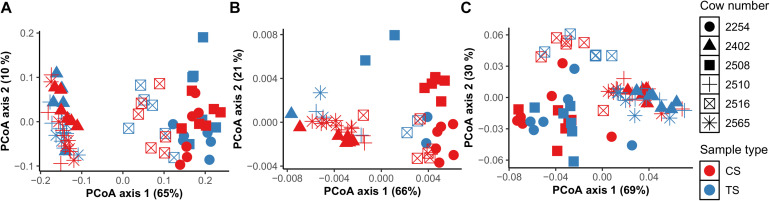
Comparison of DNA-based bacterial **(A)** and archaeal **(B)** community composition between sample types TS (tube solid) and CS (cannula solid), and between individual cows using principal coordinate analysis (PCoA) based on weighted UniFrac distances. PCoA based on Bray Curtis distances using VFA profiles **(C)** between sample types TS (tube solid) and CS (cannula solid) and between individual cows.

To investigate bacteria and archaea in the rumen, we first compared bacterial and archaeal diversity at the community and individual taxa level across TS and CS sample sites at multiple time points in 6 individual dairy cows. There were no differences between CS and TS samples for either bacteria (*P* = 0.2) or archaea (*P* = 0.1) communities when using unweighted UniFrac analysis (Supplementary Table 2). Based on individual taxonomic data, we found that TS and CS samples had commonly present bacteria and archaeal populations, indicating the presence of a core population of bacteria and archaea in these rumen samples (Supplementary Table 3). At the community level, in both CS and TS samples, similar clustering patterns were observed for bacteria and archaea. Interestingly, these clustering patterns occurred by individual cows and not by sampling times (Figures 1A,B).

### cDNA-Based Bacterial and Archaeal Communities Differ From DNA-Based Communities

Our next objective was to determine whether there was a difference in total (DNA-based) and potentially metabolically active (cDNA-based) components of bacterial and archaeal communities in TS and CS samples. For bacteria, lower species richness and Shannon diversity were observed (*P* < 0.05) for cDNA-based compared to total components in both CS and TS samples (Supplementary Figures 1, 2). A reverse pattern was observed for archaea where higher species richness and Shannon diversity were noted for cDNA-based than total components. Notably, differences in observed species and Shannon diversity in both cDNA-based and total components were observed between individual cows but not between sampling times (Supplementary Table 4).

**FIGURE 2:**
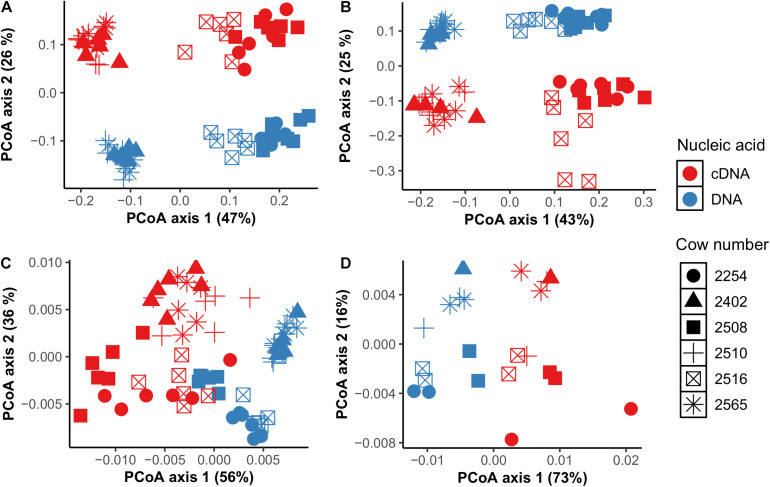
Comparison of total (DNA-based) and potentially metabolically active (cDNA-based) bacterial **(A,B)** and archaeal **(C,D)** communities using principal coordinate analysis based on weighted UniFrac distances in CS (cannula solid; **A,C)** and TS (tube solid; **B,D)** samples.

At the individual bacterial population level, we found a total of 36 genera in CS samples and 42 genera in TS samples that were different between cDNA-based and total components including unclassified *Prevotellaceae*, unclassified *Clostridiales*, *Ruminococcus*, and unclassified *Succinivibrionaceae* (*P* < 0.05) (Supplementary Table 5). *Ruminococcus* was three times higher and unclassified *Succinivibrionaceae* was 13 times higher in the cDNA-based component than the total component in CS samples. *Prevotella* (28.2% vs. 23.8%) and unclassified *Clostridiales* (15.5% vs. 13.5%) were higher in the total bacterial component compared to the cDNA-based component in CS samples. Among archaea, *Methanosphaera* was twice as high in the cDNA-based component than in the total component of CS samples (11.2% vs. 5.9%), but *Methanobrevibacter* was higher in the total component compared to the cDNA-based component in CS samples (94.0% vs. 88.6%) (*P* < 0.05) (Supplementary Table 5). Based on weighted and unweighted UniFrac distance matrix analysis, we observed that clustering of communities in both total and cDNA-based components was driven by individual cows but not by sampling time (Figure 2).

### Specific Potential Metabolic Associations Between Bacteria and Archaea in the Rumen

**FIGURE 3:**
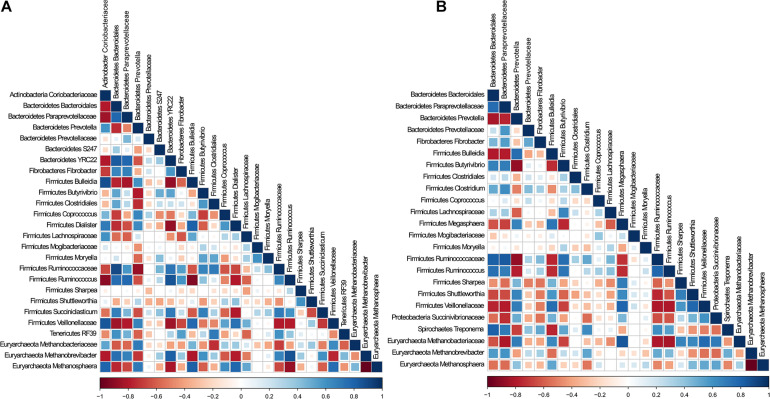
Analysis of association patterns among microbial lineages scored using Spearman correlation for **(A)** DNA and **(B)** cDNA. Individual taxa were considered present in a sample if their sequence proportion was at least 0.01% of relative abundance. Correlations are shown by the color code (blue: positive correlations, red: negative correlations).

To determine whether there were specific potential metabolic associations between bacteria and archaea, and whether these cohorts differed between cDNA-based and total components, we selected the predominant bacteria and archaea populations in CS and TS samples and performed Spearman correlation analysis. Interestingly, both TS and CS samples revealed similar correlation coefficient patterns (Supplementary Figure 3). The correlation analysis was performed individually for total and cDNA-based components (Figure 3). In both components, *Methanobrevibacter* was positively associated with *Ruminococcus*, unclassified *Ruminococcaceae*, and *Prevotella*; unclassified *Methanobacteriaceae* was positively associated with *Bulleidia* and *Prevotella*; and *Methanosphaera* was negatively associated with unclassified *Paraprevotellaceae* and *Ruminococcus*. Notably, in both components, *Methanobrevibacter* was negatively associated with *Methanosphaera*. *Methanobrevibacter* was strongly negatively correlated with unclassified *Lachnospiraceae*, *Coprococcus*, and *Dialister* in the total component. These connections were weakly negatively correlated in the cDNA-based component. In the cDNA-based component, *Methanosphaera* was positively correlated with unclassified *Succinivibrionaceae*, a correlation that was not noted in the total component.

### Correlations Between cDNA-Based Bacterial Genera and VFA Are Host-Specific

**FIGURE 4:**
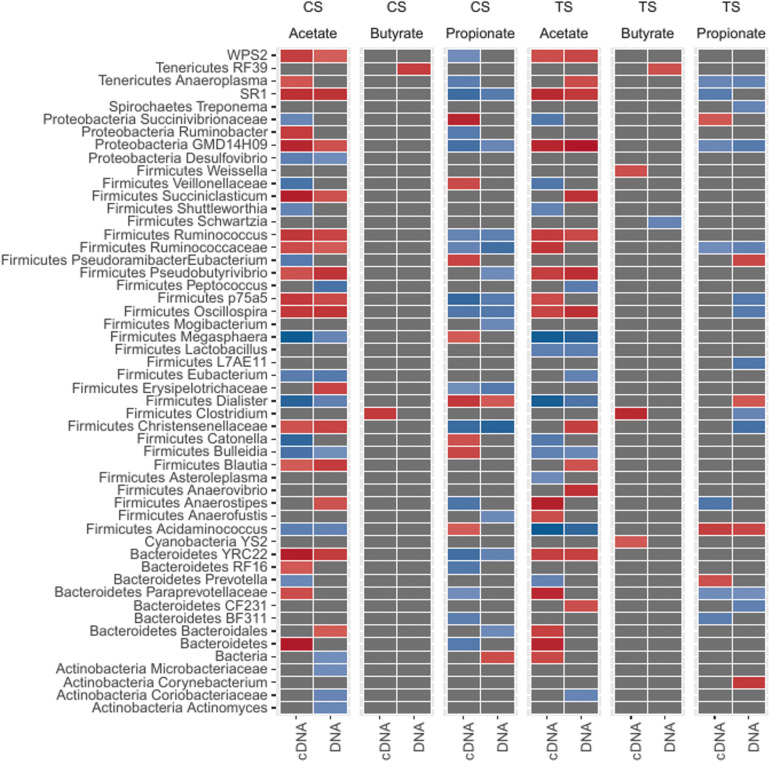
Association pattern between bacterial genera and VFA (volatile fatty acids; acetate, butyrate, and propionate) based on Spearman’s correlations. Abundant bacterial taxa were selected (ANCOM test) and correlation coefficients (r) greater than 0.6 (+ve or –ve) and *P* ≤ 0.01 were considered significant. The red color indicates positive correlations (ranged from 0.6 to 0.8). The blue color indicates negative correlations (ranged from –0.6 to –0.8).

We next sought to understand which bacteria were most closely associated with the major VFA (acetate, butyrate, and propionate; Supplementary Table 6) in the rumen. To this end, we performed correlation analysis (Figure 4; Spearman’s correlations) between total bacteria and VFA and between cDNA-based bacteria and VFA, and also compared the correlations to each other. Results showing positive and negative correlations between total and cDNA-based bacteria and individual VFA are illustrated in Figure 4.

A total of 42 genera from CS and TS samples were correlated with acetate, while 36 genera from CS and TS samples were correlated with propionate (*P* < 0.05). Only five genera were correlated with butyrate in either sample type (Figure 4). Correlations between bacterial ASV (species) and VFA are presented in Supplementary Table 7A. Approximately 1,300 ASV were found to be correlated (Spearman correlation; *r* > 0.6 and *P* < 0.05) with any one of the VFA across both sample types and DNA/cDNA communities.

In both components, from CS and TS samples, *Ruminococcus*, unclassified *Ruminococcaceae*, *Blautia*, and *YRC22* were positively correlated with acetate, while *Ruminococcus*, unclassified *Ruminococcaceae*, and *YRC22* were negatively correlated with propionate. The correlations that were noted in the cDNA-based component only included unclassified *Succinivibrionaceae* with acetate and propionate, *Prevotella* with acetate and propionate, *Bulleidia* with propionate, and *Clostridium* with butyrate. As there were several ASV identified within *Ruminococcus*, *Prevotella*, and *Succinivibrionaceae*, we performed correlations between the ASV of these genera and VFA (Supplementary Table 7B). The most abundant ASV within each of the genera contributed to more than 70% of relative abundance of each genus. Notably, the species remained unclassified and followed the same correlation patterns as were observed at the genus level. For the ASV where species was identified the relative abundance was lower (<0.1% of total bacterial abundance) and the correlation values were below the Spearman correlation (r) threshold.

Using VFA data from all cows, principal coordinates were derived to identify clustering patterns among cows (Figure 1C) to determine whether host specificity would be observed for molar proportions of VFA. Indeed, we found that molar proportions of VFA were host-specific and that the community clustering patterns were similar for bacteria, archaea, and VFA revealing that bacteria, archaea, and VFA are connected and are host-specific (Figure 1C).

### Functionalities of Potential Microbial Metabolic Interactions and Host Specificity

In both TS and CS samples, bacteria and archaea in both total and cDNA-based components and individual molar proportions of VFA were similar within each cow over 24 h but differed between cows. We observed that three cows were predominated by *Ruminococcus* bacteria and *Methanobrevibacter* archaea, two cows were predominated by unclassified *Succinivibrionaceae* and *Prevotella* bacteria and higher *Methanosphaera* archaea, and one cow had higher unclassified *Clostridiales* bacteria and slightly higher unclassified *Methanobacteriaceae* archaea compared to other cows (Table 1). Notably, these three groups of cows matched host specificity for individual VFA. The first cluster, which was predominated by *Ruminococcus* bacteria and *Methanobrevibacter* archaea, had higher acetate proportions compared to the other three cows. The second cluster, which was predominated by unclassified *Succinivibrionaceae* bacteria and had higher proportions of *Methanosphaera* archaea, had higher concentrations of propionate compared with the other four cows. The third cluster had only one cow, which produced higher butyrate and had higher unclassified *Clostridiales* bacteria and slightly higher unclassified *Methanobacteriaceae* archaea compared to other cows. These results indicate specific bacteria-archaea cohorts that differed between individual cows, forming three potential metabolic cohorts and further suggesting an associated relationship between these cohorts and the specific molar proportions of VFA in the rumen.

**TABLE 1:** Relative abundance (%) of the most abundant cDNA-derived bacteria and archaea at the genus level in the rumen of cows identified within each cluster from cannula solid (CS) samples.

	**Acetate cows–cluster 1**			**Propionate cows–cluster 2**		**Butyrate cow–cluster 3**
**Cow #**	**2402**	**2510**	**2565**	**2508**	**2254**	**2516**
*Prevotella*	16.7	15.6	16.1	36.0	33.0	25.4
*Fibrobacter*	1.3	3.1	2.8	1.8	1.3	2.0
*Ruminococcus*	19.8	17.7	19.7	5.3	8.0	7.0
*Clostridiales*	12.4	15.1	13.3	12.2	12.9	14.8
*Lachnospiraceae*	11.3	13.0	11.1	11.1	10.1	8.1
*Butyrivibrio*	10.8	9.5	9.7	5.9	5.7	6.3
*Ruminococcaceae*	4.1	4.5	4.4	1.4	1.6	2.4
*Succinivibrionaceae*	3.3	2.7	5.0	7.5	8.3	4.6
*Coprococcus*	3.2	2.6	3.3	3.3	2.4	2.4
*Methanobrevibacter*	88.8	90.8	90.7	83.5	87.6	90.3
*Methanosphaera*	11.1	9.2	9.2	16.4	12.0	9.4

*Three clusters of cows identified based on bacteria-archaea communities and predominant volatile fatty acid (VFA) proportions. Genera were considered present in a sample if its relative abundance was greater than 1% and it was found across 75% of samples.*

### Modeling of Functionalities of Potential Microbial Metabolic Interactions

**FIGURE 5:**
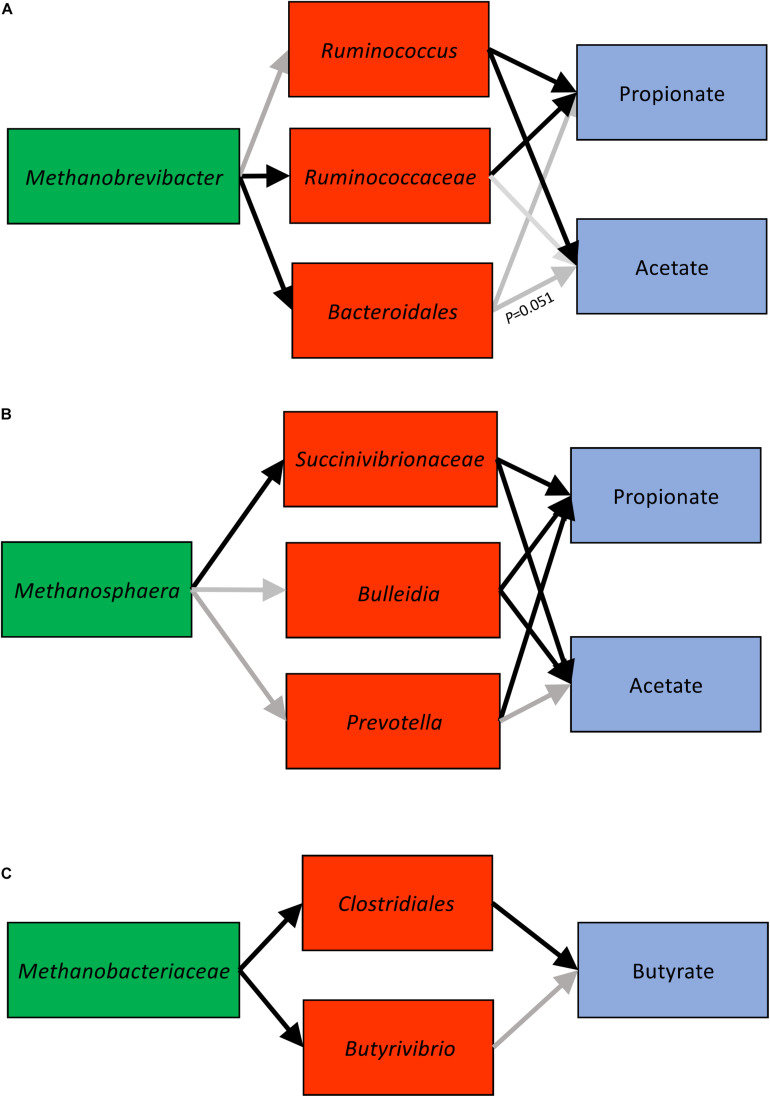
Cluster 1 **(A)**, cluster 2 **(B)**, and cluster 3 **(C)** connections derived using SEM (structural equations modeling). Black arrows indicate significant associations (*P* < 0.05).

In light of the three clusters we observed, we further used SEM techniques to determine the significance of interactions among microbial networks by using two-sided hypothesis-driven tests (Table 1 and Figure 5). Analysis of *Methanobrevibacter* cluster associations supported the expected correlations. The association of *Methanobrevibacter* with unclassified *Ruminococcaceae* and unclassified *Bacteroidales* was significant (*P* < 0.05). *Ruminococcus* had a positive significant association with acetate and a negative significant correlation with propionate (Supplementary Table 8A). The validation showed that methylotrophic *Methanosphaera* was significantly associated with unclassified *Succinivibrionaceae* (*P* < 0.05). Unclassified *Succinivibrionaceae* had a significant positive association with propionate and an inverse association with acetate (*P* < 0.05) (Supplementary Table 8B). Analysis of the third association with one cow in consideration showed significant associations between unclassified *Clostridiales* and butyrate (*P* < 0.05) (Supplementary Table 8C).

## Discussion

Microbial syntrophy is a characteristic feature of microbial communities that enables a community to survive in conditions where individual members could not (Libby et al., 2019). Methanogenic archaea play a key role in anaerobic ecosystems, and represent the most important member for effective degradation of organic matter and methane production (Moissl-Eichinger et al., 2018). Understanding the basis for the evolution of methanogens and their interactions with other microbes in a community is of paramount importance if effective CH_4_ mitigation strategies are sought (Huang et al., 2018). In this study, we identified specific potential microbial metabolic interactions between bacteria and methanogenic archaea in the complex RME of dairy cows. Not only were specific potential microbial metabolic interactions identified, we also discovered that these microbial cohorts were linked to molar proportions of VFA and that these linkages were host-specific allowing grouping of cows based on potential microbial metabolic interactions.

The inclusion of both total and potentially metabolically active microbes in this study allowed for identification of novel combinations of potential microbial metabolic networks in the RME. It has been shown that rRNA concentrations were strongly correlated with microbial activity in the sample (Lee and Kemp, 1994), and that RNA retrieved from active microbial cells is more discriminatory than using DNA, which retrieves DNA from dead and inactive cells as well as from active microbial cells (Lettat and Benchaar, 2013). Further, cDNA (RNA)-based methods are considered as proxies for metabolically active microbes and have been routinely used in rumen microbial diversity comparisons (Lettat and Benchaar, 2013; Zhu et al., 2017**;** Bailoni et al., 2021). Species in microbial communities that were previously undetected using DNA-based methods may be detected using RNA-based methods; thus, the latter method has the potential to characterize distinct phylotypes (Latham and Wolin, 1977**;** Kang et al., 2013). Our results showed clear differences between the total and cDNA-based microbial components for both bacterial and archaeal communities and revealed that less abundant bacteria such as unclassified *Succinivibrionaceae* and methanogens such as *Methanosphaera* may have a greater metabolic contribution compared to their population density. Moreover, the greater species richness and diversity in the cDNA-based archaeal community than in the total archaeal community implies that there are archaeal taxa that may be detected in the cDNA-based component only. Notably, we found a strong correlation between the less abundant but potentially more metabolically active microbes such as unclassified *Succinivibrionaceae* and *Methanosphaera* based on correlation analysis. We also found that the genus *Ruminococcus* was potentially more metabolically active than its population density would suggest. While rRNA is increasingly being used to express metabolic activity of microbes, an increase in rRNA abundance is not always correlated with microbial growth and can also happen in non-growth activities such as being in a dormant state (Blazewicz et al., 2013). In addition, staining microbial cells with 5-cyano-2,3-ditolyl tetrazolium chloride is also a commonly used method to indicate metabolic activity (Bowsher et al., 2019). Both 16S rRNA/cDNA and staining methods have advantages and limitations and should be used with caution when implications of functional potential are made based on taxonomic data. Collectively it can be inferred that while RNA-based microbial investigations provide novel insights into less abundant but potentially metabolically significant microbes, both approaches when combined can be powerful to understand the complex interactions of the RME and its interactions with the ruminant host.

Syntrophy allows a consortium of microorganisms to gain energy by coupling processes that can, due to bioenergetic reasons, be accomplished only by microbial interlinkage (Moissl-Eichinger et al., 2018). Establishment of combined metabolic activity of microorganisms depends on the activity of methanogenic archaea, who are primarily responsible for the efficient removal of H_2_ and formate–major electron carriers–in the absence of other terminal electron acceptors. In the RME, methanogens need fermenting bacteria for the production of their substantial metabolic products (Morris et al., 2013) and to maintain the lower partial pressure of H_2_ (PH_2_) in the rumen which determines redox potential and consequently influences the metabolic activity of microbes and, ultimately, feed fermentation in the rumen (Huang et al., 2018). In this study, we found three bacteria-archaea clusters that were linked to specific molar proportions of VFA in the rumen. In cluster 1, *Methanobrevibacter* was positively correlated with *Ruminococcus*, unclassified *Ruminococcaceae*, and unclassified *Bacteroidales* and these populations were positively correlated with acetate and negatively associated with propionate. These connections were unique to a cohort of dairy cows and constant with time.

The interactions between *Methanobrevibacter ruminantium* and species of *Ruminococcus* have long been established using *in vitro* experiments. For example, *Ruminococcus albus* produces more acetate and H_2_ when co-cultured with *M. ruminantium* whereas it produces acetate and ethanol when grown as a monoculture (Neill et al., 1978; Neumann et al., 1999). Similarly, *Ruminococcus flavefaciens* produces more acetate and H_2_ when co-cultured with *M. ruminantium* whereas it produces acetate and succinate when grown as a monoculture (Latham and Wolin, 1977). The interaction between species of *Ruminococcus* and *Methanobrevibacter* and their positive association with acetate as observed in the current *in vivo* experiment agrees with findings from *in vitro* studies. Fitting the cluster 1 information, i.e., the relative abundance of *Ruminococcus*, unclassified *Ruminococcaceae*, and *Methanobrevibacter* and VFA proportions, into the SEM model revealed that *Ruminococcus* was positively correlated with *Methanobrevibacter* and negatively correlated with propionate. The interaction between *Ruminococcus* and *Methanobrevibacter* was not significant but a positive correlation of unclassified *Ruminococcaceae* with *Methanobrevibacter* was identified. Species of *Ruminococcaceae* are important fibrolytic bacteria in the RME, observed in the gut from birth into adulthood (Jami et al., 2013). The functions of these species can vary depending on their interactions with methanogens as their genomes can sense H_2_ concentrations in the rumen and consequently regulate H_2_ production and alter formation of fermentation end products (Zheng et al., 2015). The limitation associated with this cluster is the lack of resolution of some lineages of *Ruminococcaceae* to the genus or species level thus not providing a greater confidence value in quantifying relationships. Future studies should isolate and characterize different species of *Ruminococcus* or rely on metagenomic information to improve resolution to species level to better understand interactions between its species and *Methanobrevibacter*. Nevertheless, this study provides the basis for hypothesis-driven (functionality) microbial clusters to test the two-way interactions between potential bacteria-archaea metabolic interactions and VFA in a SEM model.

We present interactions between *Methanosphaera* and unclassified *Succinivibrionaceae*, *Prevotella*, and *Bulleidia* as potential microbial metabolic cluster 2 cows, which is positively correlated with propionate and negatively correlated with acetate. *Methanosphaera* is methanol-reducing and has a low threshold for H_2_ and thus can outcompete other methanogens for H_2_ (Thauer et al., 2008). This might explain why *Methanosphaera* was strongly negatively correlated with *Methanobrevibacter* in this study. The dependence of *Methanosphaera* on bacteria or protozoa is not only for H_2_ but also for methanol as the latter is not a common byproduct found in the RME. The source of methanol in the rumen has been described as pectin or choline which constitute a small proportion of the ruminant diet (Weimer and Zeikus, 1977; Pozuelo et al., 2015). Feeding ensiled materials to dairy cows is a common practice in intensively managed operations which can lead to the production of ethanol and methanol which may explain the presence of *Methanosphaera* in the RME of dairy cows (Kelly et al., 2019). The link between *Methanosphaera* and *Prevotella* and unclassified *Succinivibrionaceae* has not been elucidated *in vivo*, but a possible explanation could be the production of succinate from fumarate by *Succinivibrionaceae*, which is utilized by *Prevotella* via the succinyl-CoA pathway to generate propionate. Although several microbes are involved in pectin degradation, pectin methyl esterases that convert pectin to methanol are abundant in *Prevotella* (Kelly et al., 2019); this may explain why *Prevotella* and *Methanosphaera* are strongly positively correlated. The abundance of *Prevotella* has been associated with higher propionate production in the rumen (Poeker et al., 2018), similar to our observation that *Prevotella* was positively correlated with propionate. When microbes and VFA were evaluated in the SEM model, the interactions between *Methanosphaera* and unclassified *Succinivibrionaceae* were highly significant and collectively the interaction of unclassified *Succinivibrionaceae* and *Prevotella* with propionate was highly significant. Identification and quantification of cluster 2 is unique to this study. Assessing the genomes of *Prevotella* and unclassified *Succinivibrionaceae* may reveal new insights on their mutual dependence, how they interact with *Methanosphaera*, and their collective role in propionate production.

We propose the third cluster as unclassified *Clostridiales-Butyrivibrio-* unclassified *Methanobacteriaceae*-butyrate. The potential metabolic interaction between *Clostridium thermocellum* and *Methanobacterium thermoautotrophicum* via formate and H_2_ was previously demonstrated *in vitro* (Weimer and Zeikus, 1977). More recently, in humans, a correlation between unclassified *Clostridiales* and unclassified *Methanobacteriaceae* and their link to butyrate was demonstrated (Pozuelo et al., 2015). Of the three clusters identified, this cluster represents weak interactions between bacteria and methanogens and their link to butyrate because the mechanistic basis underlying butyrate production and how butyrate-forming bacteria respond to changes in H_2_ concentrations in the rumen is less understood. Also, increasing the number of animals within the third cluster will strengthen the found interactions. In the RME, the genus *Methanobacterium* accounts for a small proportion of methanogenic archaea. We found several lineages that were only identified to the level of the family *Methanobacteriaceae*. Family *Methanobacteriaceae* was negatively correlated with unclassified *Clostridiales* and *Butyrivibrio* and the correlation was statistically significant in the SEM model. Unclassified *Clostridiales* was associated with butyrate. The potential role of unclassified *Clostridiales* in butyrate synthesis is well established in both ruminant and human research (Ørskov et al., 1988; Li et al., 2019). The potential role of *Methanobacterium* in butyrate production may be implied by the increase of butyrate measurements when methanogens are inhibited (Melgar et al., 2020), but additional information on butyrate synthesis and bacteria involved in butyrate production is needed to establish the connection between unclassified *Clostridiales* and *Methanobacterium* species. Although this study highlights the presence of potential microbial metabolic networks in the rumen, the cause-effect relationships within clusters have not been demonstrated. Further studies are needed to characterize their taxonomy as well as their functional potential and decipher the mechanistic basis of potential microbial syntrophy within each network. Applications of meta-omic approaches combined with *in vitro* experimental studies will enable us to understand the basis of microbial metabolic interactions among these clusters.

Finally, the most novel finding from this study was that potential microbial metabolic cluster-VFA connections were individual cow-specific, revealing that dairy cows may be grouped based on potential microbial metabolic interactions. The effect of host was more significant despite differences in sampling methods, sampling times, or DNA-based vs. cDNA-based analysis. A core heritable microbiome is known to exist in the rumen and host genetics have a strong influence in determining the microbial ecology of the rumen (Paz et al., 2016). However, the effects of host genetics have been confounded by multiple other factors such as diet, physiological status, and stage of production (Li et al., 2019) and therefore have not been delineated. We demonstrate, for the first time, that within cohorts of individual cows, potential microbial metabolic interactions are maintained and the proportion of specific bacteria, archaea, and VFA are constant with time.

Several reports indicate that particle retention time, a heritable trait (Tapio et al., 2017; Wallace et al., 2019), can affect H_2_ concentrations in the rumen (Janssen, 2010) and consequently influence ruminal methanogen diversity (Shi et al., 2014), which in turn is linked to CH_4_ emissions being repeatable and heritable (Roehe et al., 2016; Tapio et al., 2017; Wallace et al., 2019). Since it is clear that different methanogens compete for H_2_ (Morris et al., 2013), and a strong negative correlation was observed between *Methanobrevibacter* and *Methanosphaera* in this study, it is logical that different methanogens have specific interactions with H_2_-producing bacteria in the rumen thus leading to specific microbial cohorts. While this study demonstrated a sorting approach to group dairy cows based on specific microbial cohorts, the phenotypic performance such as feed efficiency and enteric CH_4_ emissions in dairy cows within and between groups sorted by specific microbial clusters remains to be determined. Sorting cows based on our found cohorts would reduce individual cow variation and offer the opportunities for further investigation including manipulation of the RME and development of prediction models for host phenotypic responses. Although this study provides insights into specific bacteria-archaea networks, there are yet several unidentified possible bacteria-archaea associations such as those between unclassified *Thermoplasmatales* and bacteria which should be investigated in the future. Further studies should also extend these findings to other ruminant species to demonstrate how these specific bacteria-archaea cohorts change based on ecological differences.

This study provides information on potential microbial metabolic associations and also proposes SEM as a statistical model to quantify interactions among microbial networks. The SEM model was validated by data obtained from a large number of dairy cows from a different herd, thus supporting findings of this study that specific potential microbial metabolic associations exist in the rumen. We recognize that some interactions were not statistically significant due to a lack of species level resolution. Nevertheless, the proposed approach, when backed by a larger number of animals combined with the use of omic tools including metagenomics and metatranscriptomics in future studies, may help better characterize underrepresented methanogenic archaea, such as unclassified *Methanomassiliicoccales* and unclassified *Methanobacteriaceae*, and allow the development of robust SEM modeling techniques to predict the functionalities of microbial networks. Further, sorting animals based on microbial networks is applicable to all ruminant species and could lead to the identification of animals that are energy efficient and generate less CH_4_, which is highly desirable for the advancement of animal agriculture. Findings from this study are broadly applicable to anoxic environments where microbial metabolic interactions form the basis for ecological and functionalities of microbiota and their interactions with the host and habitat.

## Data Availability Statement

Bacterial and archaeal raw sequences have been deposited in the NCBI Sequence Read Archive (SRA) database under BioProject accession number PRJNA630069.

## Ethics Statement

The Pennsylvania State University Animal Care and Use Committee approved all animal-related procedures used in this study.

## Author Contributions

DP and AH: conceptualization, funding acquisition, and resources. NI and DP: data curation. VK-S, MH, NI, and DP: formal analysis. BV, JB, CD, SR, AM, KN, MF, DP, and AH: investigation. BV, AF, AS, CD, SR, AM, KN, and MF: methodology. BV, CD, DP, and AH: project administration. NI: bioinformatic analysis. NI and DS: statistical analysis. VK-S, NI, MH, DP, and AH: manuscript preparation. All authors contributed to the article and approved the submitted version.

## Conflict of Interest

The authors declare that the research was conducted in the absence of any commercial or financial relationships that could be construed as a potential conflict of interest.

## Supplementary Material

Supplementary Figure 1 Measurement of bacterial community richness **(A,B)** and Shannon diversity (alpha diversity) **(C,D)** over time **(B,D)** and per individual cow **(A,C)** in total (DNA) and active (cDNA) microbial communities. Boxes represent the interquartile range (IQR), and the lines inside represent the median. Whiskers denote the lowest and highest values within 1.5 times of the IQR; dots denote the diversity index observations for each sample.

Supplementary Figure 2 Measurement of archaeal community richness **(A,B)** and Shannon diversity (alpha diversity) **(C,D)** over time **(B,D)** and per individual cow **(A,C)** in total (DNA) and potentially active (cDNA) microbial communities. Boxes represent the interquartile range (IQR), and the lines inside represent the median. Whiskers denote the lowest and highest values within 1.5 times of the IQR; dots denote the diversity index observations for each sample.

Supplementary Figure 3 Analysis of correlation among microbial lineages scored using Spearman correlation for **(A)** cannula solid (CS) samples and **(B)** tube solid (TS) samples. Microbial taxa were considered present in a sample if their sequence proportion was at least 0.01% of relative abundance. Correlation is shown by the color code (blue: positive correlation, red: negative correlation).

Supplementary Table 1 Absolute abundance of bacterial ASV **(A)** and archaeal OTU **(B)** by sample type [tube solid (TS) and cannula solid (CS)] in total (DNA) and potentially metabolically active (cDNA) communities.

Supplementary Table 2 *P* values denoting level of significance for community patterns revealed by principal coordinate analysis estimates by sample type. Non-parametric permutational multivariate ANOVA test was used to test differences by sampling type.

Supplementary Table 3 Mean relative abundance (%) of bacterial and archaeal taxa (identified to the genus level) by sample type [tube solid (TS) and cannula solid (CS)] in total (DNA) and potentially metabolically active (cDNA) communities.

Supplementary Table 4 Kruskal–Wallis Rank Sum Test analysis for the observed species and Shannon diversity, for Supplementary Figures 1, 2.

Supplementary Table 5 Differences in mean abundance of microbial taxa between total (DNA) and potentially metabolically active (cDNA) components. Significant differences in microbe composition between DNA and cDNA detected by ANCOM at a P value threshold of 0.05.

Supplementary Table 6 Concentration of volatile fatty acids (VFA) in tube solid (TS) and cannula solid (CS) samples.

Supplementary Table 7A Amplicon sequence variants (ASV) significantly associated with volatile fatty acids (VFA) in tube solid (TS) and cannula solid (CS) samples. The correlation coefficients (r) greater than 0.6 (+ve or −ve) and *P* ≤ 0.01 were considered significant.

Supplementary Table 7B Selected amplicon sequence variants (ASV) (*Prevotella*, *Ruminococcus*, and *Succinivibrionaceae*) associated with volatile fatty acids (VFA) in the tube solid (TS) and cannula solid (CS) samples. The correlation coefficients (r) greater than 0.6 (+ve or −ve) and *P* ≤ 0.01 were considered significant.

Supplementary Table 8A Cluster 1, connections derived using SEM (structural equations modeling).

Supplementary Table 8B Cluster 2, connections derived using SEM (structural equations modeling).

Supplementary Table 8C Cluster 3, connections derived using SEM (structural equations modeling).
