# Supplementary figures and images for: Using Structural Equation Modeling to Understand Interactions Between Bacterial and Archaeal Populations and Volatile Fatty Acid Proportions in the Rumen
Source: Front Microbiol. 2021 Jun 9;12:611951. doi: 10.3389/fmicb.2021.611951 (PMC8248675; doi:10.3389/fmicb.2021.611951)

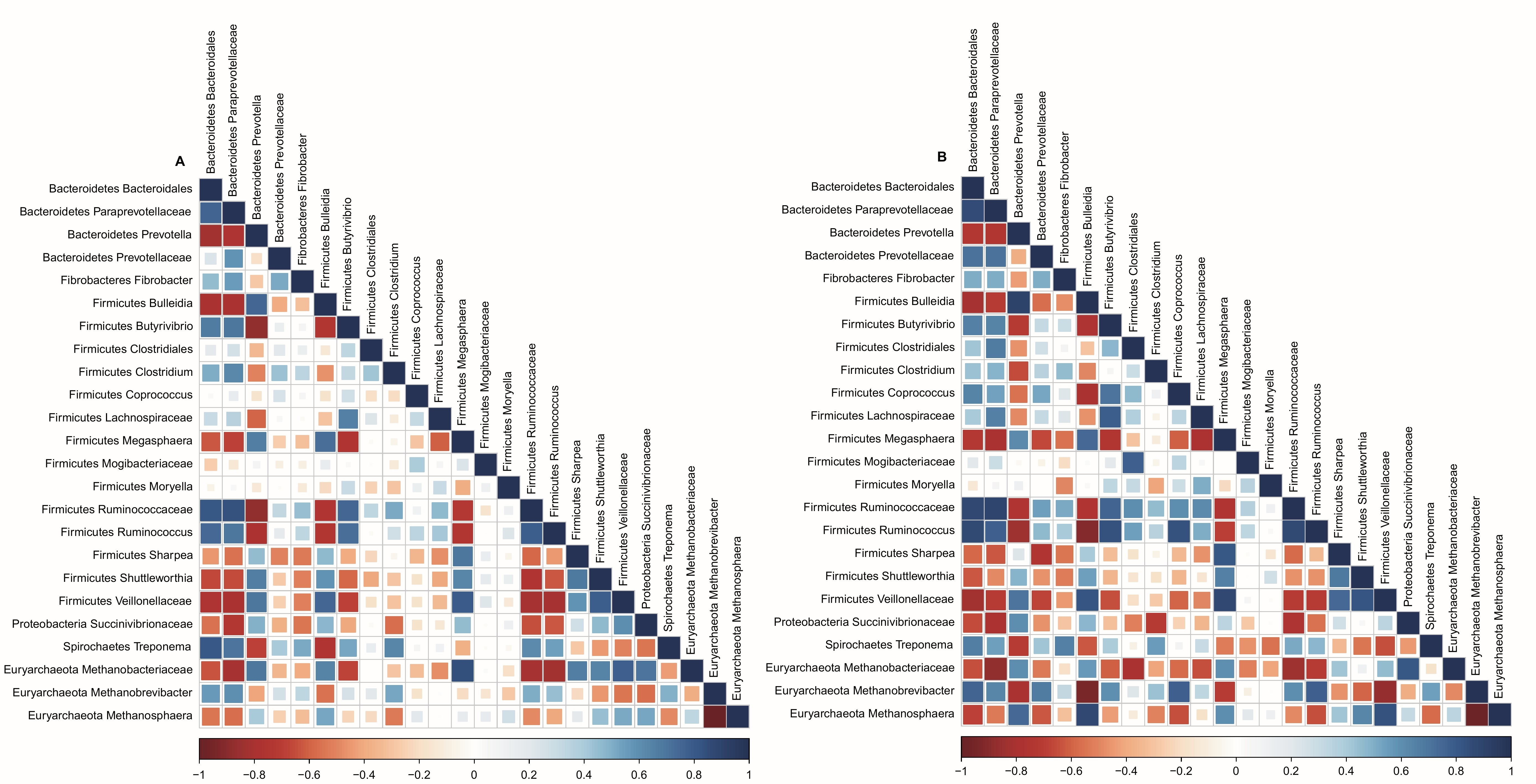

Supplement: Supplementary Figure 3 — Analysis of correlation among microbial lineages scored using Spearman correlation for (A) cannula solid (CS) samples and (B) tube solid (TS) samples. Microbial taxa were considered present in a sample if their sequence proportion was at least 0.01% of relative abundance. Correlation is shown by the color code (blue: positive correlation, red: negative correlation). [file Image_3.TIF]
