# Supplementary material for: Using Structural Equation Modeling to Understand Interactions Between Bacterial and Archaeal Populations and Volatile Fatty Acid Proportions in the Rumen
Source: Front Microbiol. 2021 Jun 9;12:611951. doi: 10.3389/fmicb.2021.611951 (PMC8248675; doi:10.3389/fmicb.2021.611951)
Supplement: Supplementary Table 2 — P values denoting level of significance for community patterns revealed by principal coordinate analysis estimates by sample type. Non-parametric permutational multivariate ANOVA test was used to test differences by sampling type. [file Table_2.DOCX]

**Table S2**. *P* values denoting level of significance for community patterns revealed by principal coordinate analysis estimates by sampling type. Nonparametric permutational multivariate ANOVA test was used to test differences by sampling type.

| Bacteria | | | |
| --- | --- | --- | --- |
| Sample type | Weighted | | Unweighted |
| TS vs CS | | 0.611 | 0.198 |
| Archaea | | | |
| Sample type | | Weighted | Unweighted |
| TS vs CS | | 0.001 | 0. 120 |
