# Supplementary material for: Using Structural Equation Modeling to Understand Interactions Between Bacterial and Archaeal Populations and Volatile Fatty Acid Proportions in the Rumen
Source: Front Microbiol. 2021 Jun 9;12:611951. doi: 10.3389/fmicb.2021.611951 (PMC8248675; doi:10.3389/fmicb.2021.611951)
Supplement: Supplementary Table 3 — Mean relative abundance (%) of bacterial and archaeal taxa (identified to the genus level) by sample type [tube solid (TS) and cannula solid (CS)] in total (DNA) and potentially metabolically active (cDNA) communities. [file Table_3.DOCX]

**Table S3.** Mean relative abundance (%) of bacterial and archaeal taxa (at genus level) by sample type (tube solid [TS] and cannula solid [CS]).

|  |  | | |  | | |
| --- | --- | --- | --- | --- | --- | --- |
|  | cDNA | | DNA | | |  |
|  | CS | TS | CS | | TS |  |
| Bacteria |  |  |  | |  |  |
| Actinobacteria Actinomyces | 0.000 | 0.000 | 0.001 | | 0.001 |  |
| Actinobacteria Actinomycetaceae | 0.000 | 0.001 | 0.002 | | 0.006 |  |
| Actinobacteria Actinomycetales | 0.001 | 0.001 | 0.003 | | 0.002 |  |
| Actinobacteria Adlercreutzia | 0.000 | 0.000 | 0.023 | | 0.023 |  |
| Actinobacteria Aeromicrobium | 0.000 | 0.000 | 0.000 | | 0.001 |  |
| Actinobacteria Atopobium | 0.056 | 0.048 | 0.090 | | 0.089 |  |
| Actinobacteria Brevibacterium | 0.000 | 0.000 | 0.000 | | 0.001 |  |
| Actinobacteria Cellulosimicrobium | 0.000 | 0.000 | 0.000 | | 0.001 |  |
| Actinobacteria Coriobacteriaceae | 0.215 | 0.187 | 1.733 | | 1.774 |  |
| Actinobacteria Corynebacterium | 0.026 | 0.016 | 0.070 | | 0.067 |  |
| Actinobacteria Dietzia | 0.000 | 0.001 | 0.000 | | 0.000 |  |
| Actinobacteria Kocuria | 0.000 | 0.001 | 0.001 | | 0.001 |  |
| Actinobacteria Microbacteriaceae | 0.000 | 0.000 | 0.001 | | 0.001 |  |
| Actinobacteria Microbacterium | 0.000 | 0.001 | 0.002 | | 0.004 |  |
| Actinobacteria Propionibacteriaceae | 0.000 | 0.001 | 0.001 | | 0.001 |  |
| Actinobacteria Propionibacterium | 0.000 | 0.000 | 0.001 | | 0.000 |  |
| Actinobacteria Rhodococcus | 0.000 | 0.000 | 0.001 | | 0.000 |  |
| Actinobacteria Slackia | 0.000 | 0.000 | 0.002 | | 0.001 |  |
| Actinobacteria Streptomyces | 0.000 | 0.000 | 0.001 | | 0.001 |  |
| Armatimonadetes RB046 | 0.000 | 0.000 | 0.001 | | 0.001 |  |
| Bacteria | 0.177 | 0.195 | 0.220 | | 0.246 |  |
| Bacteroidetes | 0.035 | 0.034 | 0.000 | | 0.001 |  |
| Bacteroidetes Bacteroidales | 1.442 | 1.191 | 3.337 | | 3.177 |  |
| Bacteroidetes BF311 | 0.019 | 0.018 | 0.040 | | 0.033 |  |
| Bacteroidetes BS11 | 0.001 | 0.000 | 0.027 | | 0.024 |  |
| Bacteroidetes CF231 | 0.258 | 0.259 | 0.435 | | 0.392 |  |
| Bacteroidetes p253418B5 | 0.001 | 0.000 | 0.001 | | 0.001 |  |
| Bacteroidetes Paludibacter | 0.007 | 0.008 | 0.004 | | 0.004 |  |
| Bacteroidetes Paraprevotellaceae | 0.990 | 0.879 | 1.485 | | 1.338 |  |
| Bacteroidetes Porphyromonadaceae | 0.000 | 0.000 | 0.000 | | 0.001 |  |
| Bacteroidetes Porphyromonas | 0.000 | 0.000 | 0.000 | | 0.000 |  |
| Bacteroidetes Prevotella | 23.798 | 26.802 | 28.255 | | 27.924 |  |
| Bacteroidetes Prevotellaceae | 0.829 | 0.815 | 1.233 | | 1.287 |  |
| Bacteroidetes RF16 | 0.252 | 0.390 | 0.050 | | 0.050 |  |
| Bacteroidetes S247 | 0.385 | 0.238 | 2.633 | | 2.423 |  |
| Bacteroidetes Sphingobacterium | 0.000 | 0.000 | 0.001 | | 0.001 |  |
| Bacteroidetes YRC22 | 0.264 | 0.234 | 0.547 | | 0.473 |  |
| Cyanobacteria Streptophyta | 0.002 | 0.009 | 0.141 | | 0.120 |  |
| Cyanobacteria YS2 | 0.092 | 0.116 | 0.075 | | 0.032 |  |
| Elusimicrobia Elusimicrobiaceae | 0.007 | 0.006 | 0.003 | | 0.003 |  |
| Elusimicrobia Endomicrobia | 0.000 | 0.004 | 0.000 | | 0.000 |  |
| Fibrobacteres Fibrobacter | 2.033 | 2.089 | 0.525 | | 0.367 |  |
| Firmicutes | 0.023 | 0.019 | 0.005 | | 0.006 |  |
| Firmicutes Acidaminococcus | 0.245 | 0.210 | 0.256 | | 0.248 |  |
| Firmicutes Anaerofustis | 0.008 | 0.008 | 0.023 | | 0.023 |  |
| Firmicutes Anaerostipes | 0.176 | 0.167 | 0.035 | | 0.036 |  |
| Firmicutes Anaerotruncus | 0.000 | 0.001 | 0.000 | | 0.000 |  |
| Firmicutes Anaerovibrio | 0.015 | 0.009 | 0.043 | | 0.057 |  |
| Firmicutes Asteroleplasma | 0.027 | 0.039 | 0.018 | | 0.016 |  |
| Firmicutes Bacillus | 0.001 | 0.003 | 0.004 | | 0.007 |  |
| Firmicutes Blautia | 0.012 | 0.012 | 0.014 | | 0.014 |  |
| Firmicutes Bulleidia | 1.107 | 1.127 | 2.076 | | 2.097 |  |
| Firmicutes Butyrivibrio | 7.973 | 6.921 | 6.732 | | 6.782 |  |
| Firmicutes Carnobacterium | 0.000 | 0.000 | 0.000 | | 0.001 |  |
| Firmicutes Catonella | 0.082 | 0.079 | 0.008 | | 0.007 |  |
| Firmicutes Christensenellaceae | 0.203 | 0.165 | 0.384 | | 0.365 |  |
| Firmicutes Clostridia | 0.003 | 0.004 | 0.005 | | 0.003 |  |
| Firmicutes Clostridiaceae | 0.001 | 0.001 | 0.000 | | 0.000 |  |
| Firmicutes Clostridiales | 13.461 | 11.858 | 15.939 | | 15.471 |  |
| Firmicutes Clostridium | 1.514 | 3.437 | 0.264 | | 0.338 |  |
| Firmicutes Coprococcus | 2.871 | 2.561 | 2.068 | | 2.458 |  |
| Firmicutes Dehalobacteriaceae | 0.000 | 0.000 | 0.001 | | 0.001 |  |
| Firmicutes Dialister | 0.471 | 0.536 | 0.852 | | 1.202 |  |
| Firmicutes Enterococcus | 0.000 | 0.001 | 0.007 | | 0.004 |  |
| Firmicutes Erysipelotrichaceae | 0.015 | 0.010 | 0.024 | | 0.025 |  |
| Firmicutes Eubacterium | 0.184 | 0.160 | 0.309 | | 0.350 |  |
| Firmicutes L7AE11 | 0.031 | 0.027 | 0.094 | | 0.098 |  |
| Firmicutes Lachnobacterium | 0.008 | 0.005 | 0.014 | | 0.010 |  |
| Firmicutes Lachnospira | 0.032 | 0.035 | 0.085 | | 0.041 |  |
| Firmicutes Lachnospiraceae | 10.774 | 9.780 | 8.237 | | 8.305 |  |
| Firmicutes Lactobacillaceae | 0.004 | 0.003 | 0.002 | | 0.005 |  |
| Firmicutes Lactobacillales | 0.000 | 0.001 | 0.001 | | 0.002 |  |
| Firmicutes Lactobacillus | 0.014 | 0.019 | 0.139 | | 0.028 |  |
| Firmicutes Lactococcus | 0.000 | 0.000 | 0.001 | | 0.001 |  |
| Firmicutes Leuconostoc | 0.000 | 0.000 | 0.000 | | 0.001 |  |
| Firmicutes Megasphaera | 0.661 | 0.462 | 0.144 | | 0.125 |  |
| Firmicutes Mitsuokella | 0.030 | 0.038 | 0.013 | | 0.008 |  |
| Firmicutes Mogibacteriaceae | 1.362 | 1.132 | 1.511 | | 1.735 |  |
| Firmicutes Mogibacterium | 0.010 | 0.011 | 0.012 | | 0.012 |  |
| Firmicutes Moryella | 0.666 | 0.556 | 0.690 | | 0.648 |  |
| Firmicutes Oribacterium | 0.020 | 0.015 | 0.044 | | 0.019 |  |
| Firmicutes Oscillospira | 0.210 | 0.204 | 0.160 | | 0.178 |  |
| Firmicutes p75a5 | 0.058 | 0.048 | 0.124 | | 0.130 |  |
| Firmicutes Paenibacillus | 0.000 | 0.002 | 0.000 | | 0.001 |  |
| Firmicutes Pediococcus | 0.000 | 0.001 | 0.003 | | 0.001 |  |
| Firmicutes Peptococcus | 0.012 | 0.009 | 0.013 | | 0.011 |  |
| Firmicutes Planococcaceae | 0.001 | 0.001 | 0.001 | | 0.002 |  |
| Firmicutes Planomicrobium | 0.000 | 0.000 | 0.000 | | 0.000 |  |
| Firmicutes Pseudobutyrivibrio | 0.118 | 0.109 | 0.167 | | 0.156 |  |
| Firmicutes Pseudoramibacter | 0.099 | 0.090 | 0.068 | | 0.075 |  |
| Firmicutes RFN20 | 0.089 | 0.101 | 0.082 | | 0.112 |  |
| Firmicutes Roseburia | 0.009 | 0.012 | 0.001 | | 0.000 |  |
| Firmicutes Ruminococcaceae | 3.058 | 2.957 | 3.924 | | 4.070 |  |
| Firmicutes Ruminococcus | 12.906 | 11.199 | 4.732 | | 5.107 |  |
| Firmicutes Rummeliibacillus | 0.000 | 0.001 | 0.000 | | 0.001 |  |
| Firmicutes Schwartzia | 0.012 | 0.013 | 0.068 | | 0.065 |  |
| Firmicutes Selenomonas | 0.475 | 0.585 | 0.271 | | 0.199 |  |
| Firmicutes Sharpea | 1.006 | 0.944 | 2.546 | | 3.193 |  |
| Firmicutes Shuttleworthia | 1.450 | 1.373 | 1.368 | | 1.422 |  |
| Firmicutes Streptococcaceae | 0.000 | 0.000 | 0.000 | | 0.001 |  |
| Firmicutes Streptococcus | 0.008 | 0.017 | 0.121 | | 0.024 |  |
| Firmicutes Succiniclasticum | 0.349 | 0.291 | 2.181 | | 2.054 |  |
| Firmicutes Veillonellaceae | 0.808 | 0.872 | 0.843 | | 0.657 |  |
| Firmicutes Weissella | 0.004 | 0.007 | 0.033 | | 0.031 |  |
| Fusobacteria Fusobacterium | 0.000 | 0.000 | 0.001 | | 0.001 |  |
| Proteobacteria | 0.001 | 0.001 | 0.000 | | 0.000 |  |
| Proteobacteria Acetobacter | 0.014 | 0.021 | 0.010 | | 0.011 |  |
| Proteobacteria Agrobacterium | 0.000 | 0.000 | 0.004 | | 0.002 |  |
| Proteobacteria Alphaproteobacteria | 0.006 | 0.007 | 0.004 | | 0.004 |  |
| Proteobacteria Brucellaceae | 0.000 | 0.000 | 0.001 | | 0.001 |  |
| Proteobacteria Burkholderiales | 0.000 | 0.000 | 0.009 | | 0.007 |  |
| Proteobacteria Campylobacter | 0.000 | 0.004 | 0.001 | | 0.005 |  |
| Proteobacteria Comamonas | 0.001 | 0.001 | 0.003 | | 0.005 |  |
| Proteobacteria Denitrobacter | 0.000 | 0.000 | 0.000 | | 0.000 |  |
| Proteobacteria Desulfobulbus | 0.009 | 0.023 | 0.004 | | 0.012 |  |
| Proteobacteria Desulfovibrio | 0.080 | 0.070 | 0.044 | | 0.016 |  |
| Proteobacteria Devosia | 0.000 | 0.000 | 0.001 | | 0.001 |  |
| Proteobacteria GMD14H09 | 0.050 | 0.050 | 0.012 | | 0.019 |  |
| Proteobacteria mitochondria | 0.000 | 0.000 | 0.005 | | 0.003 |  |
| Proteobacteria Neisseriaceae | 0.001 | 0.001 | 0.001 | | 0.001 |  |
| Proteobacteria Ochrobactrum | 0.000 | 0.000 | 0.001 | | 0.000 |  |
| Proteobacteria Oxalobacteraceae | 0.000 | 0.000 | 0.001 | | 0.000 |  |
| Proteobacteria Paracoccus | 0.000 | 0.000 | 0.001 | | 0.000 |  |
| Proteobacteria RF32 | 0.018 | 0.022 | 0.025 | | 0.016 |  |
| Proteobacteria Rhodobacter | 0.000 | 0.000 | 0.000 | | 0.001 |  |
| Proteobacteria Rickettsiales | 0.002 | 0.005 | 0.005 | | 0.005 |  |
| Proteobacteria Ruminobacter | 0.049 | 0.060 | 0.004 | | 0.001 |  |
| Proteobacteria Succinivibrio | 0.036 | 0.059 | 0.013 | | 0.005 |  |
| Proteobacteria Succinivibrionaceae | 5.239 | 6.926 | 0.396 | | 0.142 |  |
| Proteobacteria Sutterella | 0.013 | 0.008 | 0.033 | | 0.025 |  |
| Proteobacteria Xanthomonadaceae | 0.000 | 0.000 | 0.000 | | 0.000 |  |
| Proteobacteria Zea | 0.000 | 0.000 | 0.001 | | 0.001 |  |
| Spirochaetes | 0.000 | 0.001 | 0.000 | | 0.001 |  |
| Spirochaetes Sphaerochaeta | 0.005 | 0.004 | 0.006 | | 0.007 |  |
| Spirochaetes Spirochaetaceae | 0.000 | 0.001 | 0.001 | | 0.001 |  |
| Spirochaetes Treponema | 0.536 | 0.575 | 0.443 | | 0.472 |  |
| SR1 | 0.076 | 0.082 | 0.209 | | 0.218 |  |
| Synergistetes Pyramidobacter | 0.020 | 0.017 | 0.013 | | 0.008 |  |
| Synergistetes Synergistaceae | 0.000 | 0.001 | 0.000 | | 0.000 |  |
| Synergistetes TG5 | 0.000 | 0.005 | 0.000 | | 0.000 |  |
| Tenericutes | 0.001 | 0.001 | 0.001 | | 0.002 |  |
| Tenericutes Anaeroplasma | 0.067 | 0.073 | 0.041 | | 0.055 |  |
| Tenericutes Anaeroplasmataceae | 0.003 | 0.004 | 0.003 | | 0.001 |  |
| Tenericutes Mollicutes | 0.001 | 0.000 | 0.003 | | 0.001 |  |
| Tenericutes Mycoplasmataceae | 0.000 | 0.000 | 0.000 | | 0.001 |  |
| Tenericutes RF39 | 0.083 | 0.066 | 0.539 | | 0.537 |  |
| TM7 F16 | 0.056 | 0.044 | 0.419 | | 0.451 |  |
| TM7 Rs045 | 0.011 | 0.009 | 0.017 | | 0.020 |  |
| WPS2 | 0.049 | 0.053 | 0.010 | | 0.011 |  |
|  |  |  |  | |  |  |
| Archaea |  |  |  | |  |  |
| Euryarchaeota Methanimicrococcus | 0.002 | 0.005 | 0.000 | | 0.000 |  |
| Euryarchaeota Methanobacteriaceae | 0.146 | 0.315 | 0.070 | | 0.147 |  |
| Euryarchaeota Methanobacterium | 0.007 | 0.013 | 0.003 | | 0.006 |  |
| Euryarchaeota Methanobrevibacter | 88.610 | 85.203 | 94.059 | | 93.283 |  |
| Euryarchaeota Methanosphaera | 11.222 | 14.430 | 5.867 | | 6.563 |  |
| Euryarchaeota vadinCA11 | 0.013 | 0.032 | 0.001 | | 0.001 |  |
