# Supplementary material for: Using Structural Equation Modeling to Understand Interactions Between Bacterial and Archaeal Populations and Volatile Fatty Acid Proportions in the Rumen
Source: Front Microbiol. 2021 Jun 9;12:611951. doi: 10.3389/fmicb.2021.611951 (PMC8248675; doi:10.3389/fmicb.2021.611951)
Supplement: Supplementary Table 4 — Kruskal–Wallis Rank Sum Test analysis for the observed species and Shannon diversity, for Supplementary Figures 1, 2. [file Table_4.DOCX]

**Table S4:** Kruskal-Wallis Rank Sum Test analysis for the observed species and Shannon diversity, for supplementary figures S1 and S2.

|  | | Observed species | Shannon |
| --- | --- | --- | --- |
| Bacteria | | | |
| DNA | Time | 0.7754 | 0.9811 |
|  | Animal | 2.43E-05 | 2.21E-05 |
| cDNA | Time | 0.8512 | 0.875 |
|  | Animal | 1.29E-05 | 2.61E-05 |
| Archaea | | | |
| DNA | Time | 0.9159 | 0.9948 |
|  | Animal | 6.77E-06 | 6.13E-06 |
| cDNA | Time | 0.6732 | 0.6935 |
|  | Animal | 3.18E-05 | 2.65E-05 |
