# Supplementary material for: Using Structural Equation Modeling to Understand Interactions Between Bacterial and Archaeal Populations and Volatile Fatty Acid Proportions in the Rumen
Source: Front Microbiol. 2021 Jun 9;12:611951. doi: 10.3389/fmicb.2021.611951 (PMC8248675; doi:10.3389/fmicb.2021.611951)
Supplement: Supplementary Table 5 — Differences in mean abundance of microbial taxa between total (DNA) and potentially metabolically active (cDNA) components. Significant differences in microbe composition between DNA and cDNA detected by ANCOM at a P value threshold of 0.05. [file Table_5.DOCX]

**Table S5.** Differences in microbial taxa between total (DNA) and metabolically active (cDNA) components. Significant differences in microbe composition between DNA and cDNA detected by ANCOM at a *P* value threshold of 0.05.

|  | cDNA | DNA | cDNA | DNA |
| --- | --- | --- | --- | --- |
| Sampling method | CS | CS | TS | TS |
| Actinobacteria | 0.30 | 1.93 | 0.26 | 1.98 |
| Coriobacteriaceae | 0.21 | 1.73 | 0.19 | 1.77 |
| Bacteroidetes | 28.28 | 38.05 | 30.87 | 37.13 |
| Bacteroidales | 1.44 | 3.34 | 1.19 | 3.18 |
| Paraprevotellaceae | 0.99 | 1.48 | 0.88 | 1.34 |
| Prevotellaceae | 0.83 | 1.23 | 0.82 | 1.29 |
| Prevotella | 23.79 | 28.23 | 26.79 | 27.90 |
| S24-7 | 0.39 | 2.63 | 0.24 | 2.42 |
| Fibrobacteres | 2.03 | 0.52 | 2.09 | 0.37 |
| Anaerostipes | 0.18 | 0.04 | 0.17 | 0.04 |
| Bulleidia | 1.11 | 2.08 | 1.13 | 2.10 |
| Clostridiales | 13.46 | 15.94 | 11.86 | 15.47 |
| Lachnospiraceae | 10.77 | 8.24 | 9.78 | 8.31 |
| Mogibacteriaceae | 1.36 | 1.51 | 1.13 | 1.74 |
| Ruminococcaceae | 3.06 | 3.92 | 2.96 | 4.07 |
| Ruminococcus | 12.91 | 4.73 | 11.20 | 5.11 |
| Selenomonas | 0.47 | 0.27 | 0.59 | 0.20 |
| Succiniclasticum | 0.35 | 2.18 | 0.29 | 2.05 |
| Proteobacteria | 5.52 | 0.58 | 7.26 | 0.29 |
| Succinivibrio | 0.04 | 0.01 | 0.06 | 0.00 |
| Succinivibrionaceae | 5.24 | 0.40 | 6.93 | 0.14 |
| Unclassified Bacteria | 0.18 | 0.22 | 0.19 | 0.25 |
| Methanobrevibacter | 88.61 | 94.06 | 85.61 | 93.28 |
| Methanosphaera | 11.22 | 5.87 | 14.43 | 6.56 |
