# Supplementary material for: Using Structural Equation Modeling to Understand Interactions Between Bacterial and Archaeal Populations and Volatile Fatty Acid Proportions in the Rumen
Source: Front Microbiol. 2021 Jun 9;12:611951. doi: 10.3389/fmicb.2021.611951 (PMC8248675; doi:10.3389/fmicb.2021.611951)
Supplement: Supplementary Table 8A — Cluster 1, connections derived using SEM (structural equations modeling). [file Table_8.DOCX]

**Table S8A.** Cluster 1, connections derived using SEM (structural equations modeling).

| Cluster 1 | Coef. | Std. Err. | z | P>\|z\| | [95% Conf. Interval] | |
| --- | --- | --- | --- | --- | --- | --- |
| Ruminococcus |  |  |  |  |  |  |
| Methanobrevibacter | -0.233 | 0.230 | -1.010 | 0.311 | -0.683 | 0.217 |
| Intercept | 30.699 | 21.013 | 1.460 | 0.144 | -10.486 | 71.884 |
| Ruminococcaceae |  |  |  |  |  |  |
| Methanobrevibacter | 0.228 | 0.034 | 6.750 | 0.000 | 0.162 | 0.294 |
| Intercept | -17.162 | 3.095 | -5.550 | 0.000 | -23.228 | -11.096 |
| Bacteroidales |  |  |  |  |  |  |
| Methanobrevibacter | 0.284 | 0.025 | 11.290 | 0.000 | 0.235 | 0.334 |
| Intercept | -23.492 | 2.305 | -10.190 | 0.000 | -28.010 | -18.974 |
| Acetate |  |  |  |  |  |  |
| Ruminococcus | 0.254 | 0.062 | 4.070 | 0.000 | 0.132 | 0.376 |
| Ruminococcaceae | 0.592 | 0.502 | 1.180 | 0.238 | -0.392 | 1.575 |
| Bacteroidales | 0.913 | 0.468 | 1.950 | 0.051 | -0.004 | 1.831 |
| T | -0.333 | 0.069 | -4.820 | 0.000 | -0.469 | -0.198 |
| Intercept | 53.854 | 0.831 | 64.780 | 0.000 | 52.225 | 55.483 |
| Propionate |  |  |  |  |  |  |
| Ruminococcus | -0.131 | 0.049 | -2.670 | 0.008 | -0.227 | -0.035 |
| Ruminococcaceae | -1.224 | 0.394 | -3.100 | 0.002 | -1.996 | -0.451 |
| Bacteroidales | -0.238 | 0.368 | -0.650 | 0.518 | -0.958 | 0.483 |
| T | 0.120 | 0.054 | 2.200 | 0.028 | 0.013 | 0.226 |
| Intercept | 31.318 | 0.653 | 47.950 | 0.000 | 30.038 | 32.598 |

**Table S8B.** Cluster 2, connections derived using SEM (structural equations modeling).

| Cluster 2 | Coef. | Std. Err. | z | P>\|z\| | [95% Conf. Interval] | |
| --- | --- | --- | --- | --- | --- | --- |
| Prevotella |  |  |  |  |  |  |
| Methanosphaera | 0.485 | 0.268 | 1.810 | 0.071 | -0.041 | 1.010 |
| Intercept | 20.371 | 2.489 | 8.180 | 0.000 | 15.493 | 25.249 |
| Bulleidia |  |  |  |  |  |  |
| Methanosphaera | 0.064 | 0.042 | 1.520 | 0.127 | -0.018 | 0.146 |
| Intercept | 0.769 | 0.390 | 1.970 | 0.049 | 0.005 | 1.534 |
| Succinivibrionaceae |  |  |  |  |  |  |
| Methanosphaera | 0.563 | 0.050 | 11.150 | 0.000 | 0.464 | 0.661 |
| Intercept | -2.296 | 0.468 | -4.900 | 0.000 | -3.214 | -1.379 |
| Acetate |  |  |  |  |  |  |
| Prevotella | -0.098 | 0.061 | -1.610 | 0.108 | -0.218 | 0.021 |
| Bulleidia | -1.370 | 0.395 | -3.470 | 0.001 | -2.144 | -0.596 |
| Succinivibrionaceae | -0.341 | 0.118 | -2.880 | 0.004 | -0.573 | -0.109 |
| t | -0.270 | 0.083 | -3.240 | 0.001 | -0.432 | -0.107 |
| Intercept | 65.422 | 1.367 | 47.850 | 0.000 | 62.743 | 68.102 |
| Propionate |  |  |  |  |  |  |
| Prevotella | 0.187 | 0.038 | 4.890 | 0.000 | 0.112 | 0.262 |
| Bulleidia | 0.746 | 0.236 | 3.160 | 0.002 | 0.284 | 1.209 |
| Succinivibrionaceae | 0.356 | 0.071 | 4.990 | 0.000 | 0.216 | 0.497 |
| t | 0.112 | 0.051 | 2.200 | 0.028 | 0.012 | 0.212 |
| Intercept | 18.564 | 0.865 | 21.450 | 0.000 | 16.868 | 20.260 |

**Table S8C.** Cluster 3, connections derived using SEM (structural equations modeling).

| Cluster 3 | Coef. | Std. Err. | z | P>\|z\| | [95% Conf. Interval] | |
| --- | --- | --- | --- | --- | --- | --- |
| Clostridiales |  |  |  |  |  |  |
| Methanobacteriaceae | -13.805 | 5.027 | -2.750 | 0.006 | -23.658 | -3.952 |
| Intercept | 16.084 | 0.524 | 30.700 | 0.000 | 15.057 | 17.111 |
| Butyrivibrio |  |  |  |  |  |  |
| Methanobacteriaceae | -11.973 | 3.517 | -3.400 | 0.001 | -18.867 | -5.079 |
| Intercept | 8.584 | 0.367 | 23.420 | 0.000 | 7.866 | 9.303 |
| Butyrate |  |  |  |  |  |  |
| Clostridiales | 0.316 | 0.095 | 3.310 | 0.001 | 0.129 | 0.502 |
| Butyrivibrio | -0.017 | 0.134 | -0.120 | 0.901 | -0.280 | 0.246 |
| t | 0.145 | 0.069 | 2.100 | 0.036 | 0.010 | 0.280 |
| Intercept | 6.315 | 1.699 | 3.720 | 0.000 | 2.985 | 9.645 |
